# Research on China's Monetary Policy Orientation and Regulation in COVID-19

**DOI:** 10.3389/fpubh.2022.865603

**Published:** 2022-05-31

**Authors:** Baicheng Zhou, Zilun Huang, Shu Wang

**Affiliations:** ^1^China Center for Public Sector Economy Research, Jilin University, Changchun, China; ^2^School of Economics, Jilin University, Changchun, China

**Keywords:** COVID-19, Taylor rule, monetary policy, inflation, output gap, China

## Abstract

The outbreak of COVID-19 in 2019 has caused a huge impact on the global economy. In this context, it is of great significance to study the orientation and regulation of China's monetary policy, which aims to mitigate the external impact brought by COVID-19. Therefore, this paper uses the SV-TVP-FAVAR model to analyze the dynamic relationships among interest rate, inflation gap and output gap. The main conclusions are as follows. First, the output gap has a significant impact on the adjustment of the interest rate and inflation gap. In the COVID-19 era, the former response is positive and the latter response is negative. Second, the impact of the inflation gap on the interest rate fluctuates frequently, but the impact has gradually weakened in recent years. In addition, the inflation gap shows a significant positive response to the impact of the output gap. Third, interest rate is characterized by targeting the output gap and the inflation gap in the short term. However, in the period of COVID-19, the regulation effect of China's monetary policy on the inflation gap and the output gap has weakened. Meanwhile, compared with targeting the output gap, monetary policy has a more obvious orientation to control inflation.

## Introduction

The outbreak of COVID-19 in late December 2019, a sudden public health event, has caused a significant impact on the world. At present, the COVID-19 continues to spread globally with unpredictable variations. Among them, the Omicron strain has replaced the Delta strain as the main epidemic strain in the world due to its strong transmission. According to the WHO, as of February 18, 2022, the number of confirmed COVID-19 cases in the world had increased by 1,963,747 from the previous day to 4,180,650,474. The number of deaths had increased to 5,866,224. In this context, many countries have chosen to implement or extend COVID-19 prevention and control measures (1, 2), which have restricted turnover, labor supply, consumption, investment, imports, and exports.

The uncertainty of the epidemic is a major factor affecting the economic prospect. The IMF pointed out in the Global Financial Stability Report that COVID-19 has caused an economic recession and further aggravated financial fragility. Economic recovery has been hampered by the highly contagious COVID-19 pandemic. Under the background of globalization, China is also facing the challenge of economic recovery (3), The central bank's monetary policy, as an important economic means, should play a role for the state to carry out macro-control and guide market expectations. In the post-COVID-19 era, economic recovery and its sustainability are facing challenges. China's monetary policy should strike a balance among the multiple goals of inflation, economic development, and fighting the epidemic. Therefore, we need to rethink and evaluate China's monetary policy.

In order to alleviate the external shocks brought by COVID-19 and iron out short-term economic fluctuations, China has chosen to adopt a prudent and flexible monetary policy. In retrospect, China implemented a prudent monetary policy and maintained its stability and continuity after the outbreak of SARS in 2003, thus stimulating economic growth. In response to the global financial crisis, China had adopted a moderately easy monetary policy since the third quarter of 2008. It can be seen that the monetary policy adopted by China to deal with the crisis is flexible and targeted and is characterized by “leaning against the wind”. However, we need to understand the reasons and mechanism behind the characteristics of monetary policy implementation, that is, how monetary policy coordinates with output and inflation. On this basis, we further pay attention to the crisis periods. By comparing the SARS period and the global financial crisis period, this paper analyzes the particularity of China's monetary policy orientation and regulation mode during the COVID-19 period, and studies whether the monetary policy is more focused on inflation or economic growth. It is also conducive to the central bank to judge the transmission of policy signals and grasp the operation of the macroeconomy.

Given this, based on the traditional linear monetary policy, this paper attempts to construct a non-linear model in the macroeconomic system that can reflect the dynamic time-varying relationships among monetary policy tools, output gap, and inflation gap. Then it identifies the trajectory of China's monetary policy implementation mode and analyzes the dynamic feedback mechanism of monetary policy to the macroeconomy. It can be seen that compared with the existing literature, the main contributions of this paper are reflected in the following aspects: First, a time-varying parameter econometric model is constructed, which can identify the macroeconomic regulation mode of monetary policy. It can also effectively capture time-varying shock forms of monetary policy tools, output, and inflation under different economic backgrounds. In particular, a more accurate wavelet filtering method is adopted to measure the output gap (4). Second, through combing the historical evolution of China's monetary policy orientation, a background data set is constructed with a large number of macroeconomic variables related to the implementation of monetary policy. That can avoid the problem of distortion of empirical results caused by the loss of important variables as much as possible. Third, we not only identify the dynamic trajectory of monetary policy, but also use the innovative random walk method to deal with monetary policy parameters, and reversely deduce the three-dimensional impact effect of time-varying monetary policy with Bayesian estimation based on Markov Chain Monte Carlo (MCMC) method. We then effectively analyze the monetary policy tools under the impact of COVID-19's economic system. On this basis, the dynamic adjustment mechanism among monetary policy tools, output gap, and inflation gap is effectively analyzed when the economic system encounters severe negative shocks under the impact of COVID-19. A new understanding of the dynamic characteristics of monetary policy macro-control can be regarded as an important supplement to the current empirical research on monetary policy. In conclusion, a new understanding of the dynamic characteristics of macroeconomic regulation of monetary policy can be established, which can be regarded as an important complement to the current empirical research on monetary policy.

The rest of the paper is structured as follows. The second part is the literature review. The third part describes the SV-TVP-FAVAR model. The fourth part is the selection and description of the variables. The fifth part is an empirical result analysis. We study the dynamic relationships among variables by drawing the impulse response function images. And select three special periods for comparison, focusing on the orientation and regulation of China's monetary policy in the post-epidemic era. The last part gives corresponding conclusions and policy recommendations for analysis results.

## Literature Review

There has always been a debate over adopting the discretionary or rule-based monetary policy. In 1929, the outbreak of the capitalist economic crisis led to the Great Depression. Given this, Keynes (5) creatively proposed the discretionary monetary policy, arguing that the central bank should adopt different monetary policies according to the actual economic situation. However, with the emergence of stagflation, the problems existing in discretionary choice are gradually revealed. The monetarist school concluded that discretion ignored the phenomenon of time lag of monetary policy, which also produced the problem of dynamic inconsistency, thus damaging social welfare and affecting economic stability (6–8). Therefore, they advocated implementing rule-based monetary policy, mainly the Friedman rule, McCallum rule, and Taylor Rule (9–11). Among them, the Taylor rule has received widespread attention. Taylor rule is a short-term interest rate adjustment policy based on inflation and output that has the characteristics of discretionary and flexible adjustment and belongs to the standard monetary policy category, which effectively integrates the two seemingly opposite economic policies. On this basis, this paper takes the Taylor rule as the main object to study the preference and regulation mode of China's monetary policy.

However, with the deepening of the research, it is difficult to unify the linear and static Taylor rule with reality. That is to say, in the complex fact, the adjustment of inflation and output gap by this interest rate rule may be insufficient (12). Hence, since the Taylor rule was put forward, many researchers have expanded it and studied its applicability from various aspects. First of all, Taylor (13) and Bec et al. (14) pointed out that under the influence of policies, economic cycles, and other factors, the response coefficients of interest rate to inflation and output gap differed in different periods. It is contrary to the traditional linear Taylor rule. Many scholars have carried out more in-depth studies on the non-linear characteristics of the Taylor rule in practical application from different perspectives. Nobay and Peel (15), Ruge-Murcia (16), Dolado et al. (17), and Tillmann (18), respectively analyzed the causes of non-linear Taylor rule from the aspects of asymmetric central bank preferences, aggregate supply curve non-linearity, and parameter uncertainty. In addition, considering the structural changes of monetary policy, Primiceri (19) proposed a time-varying parameter vector autoregression model and used the MCMC method for estimation, believing that the Taylor rule has time-varying characteristics. Nguyen et al. (20) identified that the non-linear Taylor rule with time-varying parameters could better measure the monetary policy behavior of the United States after the 1960s.

In recent years, China has begun to focus more on studying and improving the Taylor rule to capture better the interaction among interest rate, inflation, and the output gap. Xie and Luo (21) applied Taylor's rule to China's monetary policy for the first time and found that Taylor's management can better measure China's economic policy. Wang (22) used the monetary policy response function to estimate Taylor's rule. They believed that China's monetary policy could better reflect inflation changes but had no response to the output gap. However, with the advancement of research, some scholars hold different opinions. For example, Liu and Zhang (23) used the HTSTR method to point out that the central bank's monetary policy has non-linear characteristics and asymmetry of avoiding economic contraction and preferring inflation. After considering the non-linear and time-varying factors in applying the Taylor rule, Chen et al. (24) used the TVP-SVAR model to analyze and conclude that China's monetary policy fixed on the output gap and inflation. By constructing two-stage Bayesian joint estimation, Liu and Zhang (25) believed that the monetary policy preferences of central banks were time-varying and asymmetrical. Based on the analyzed reasons and thought, this behavior was affected by economic fluctuations, monetary policy cycles, economic policy uncertainties, and preference substitution effect. Chen et al. (26) and Zhang and Liu (27) further improve the central bank's preferred behavior.

Although significant progress has been made in studying central banks' monetary policy rules and orientation, the above literature ignores the adoption of different output gap measurement methods. The results may be materially different. Therefore, the effectiveness of the output gap has a significant impact on the formulation and implementation of monetary policies and the performance of the macroeconomy (28, 29). Output gap represents the difference between potential output and actual output. Currently, the measurement methods of the output gap can be divided into three types (30). The first type is time-domain analysis, which analyzes the structural characteristics of data changes over time, mainly represented by the production function approach and SVAR method. The second type: frequency domain analysis, such as the Hodrick-Prescott and Baxter-King filter. The third type is the time and frequency domain combined methods, such as wavelet filtering and singular spectrum analysis methods. On this basis, to improve the study's accuracy, this paper selects four ways in the above types, namely production function method, Hodrick-Prescott filter, wavelet filtering method, and singular spectrum analysis method for comparison. It selects one of the four methods in line with China's national conditions.

To sum up, scholars at home and abroad have made significant progress in the improvement and orientation of monetary policy through exploration and research, which also provides a good reference for the study of this paper. However, there are still several deficiencies in current studies. First, most literatures choose HP filter method to measure the output gap when studying Taylor rule. Still, different measurement methods will produce specific results, affecting policy implementation. Secondly, although the non-linear and structural changes among variables have been considered in the macroeconomic system structure in recent years, the economic variables included in the model are relatively few, and it is difficult to depict the complex reality. Thirdly, few studies have focused on the changing monetary policy orientation and preference of central banks in the post-covid-19 era. Based on these problems, the main contributions of this paper are reflected in the following three aspects: Firstly, Taylor's rule is used to describe China's monetary policy. After the reliability test of different output gap measurement methods, it is found that the wavelet filtering method can measure China's output gap most accurately. Secondly, as the macroeconomic environment is constantly changing, China's economic structure and monetary policy are also constantly adjusting. If we simply analyze several variables related to monetary policy, it cannot cover the complex economic situation. To avoid the lack of information in the economic system, this paper uses the extension of the VAR model– factor-augmented vector autoregressive model with time-varying parameters and stochastic volatility (SV-TVP-FAVAR) model. Public factors are proposed in many macroeconomic variables. This model not only considers the endogeneity and time-varying characteristics of variables, but also absorbs the advantages of factor analysis. Common factors are proposed in a large number of macroeconomic variables. The extracted unobservable factors are used to replace information in the macro data background. Therefore, it can capture the structural changes in the economic system, so as to more accurately measure the dynamic change trend and characteristics caused by monetary policy on macroeconomic changes. Finally, because of the significant impact of the outbreak of COVID-19 on the economy and finance, this article takes the post-COVID-19 era as the research background. The monetary policy parameters are processed using an innovative random walk method, and the three-dimensional time-varying shock effect of the monetary policy is reversely derived with Bayesian estimation based on Markov Chain Monte Carlo (MCMC) method. We should study the orientation and regulation mode of the central bank's monetary policy during this period to determine whether it is more focused on the inflation gap or output gap, and analyze the causes. It can help the monetary policy better identify its location, enhance its pertinence and flexibility, and give play to its guiding and regulating role.

## Model Specification

### Basic Theoretical Analysis

According to Taylor's setting, the central bank will adjust the short-term nominal interest rate according to the output gap and inflation gap, so the generalized “Taylor rule” monetary policy form is as follows:


(1) $$i_{t}^{*}={\bar{i}}_{0}+\beta (\pi_{t}-\pi_{t}^{*})+\gamma y_{t}$$

Where $i_{t}^{*}$ represents the short-term nominal interest rate recommended by Taylor rule, ${\bar{i}}_{0}$ represents the long-term and time-varying equilibrium interest rate, $\pi_{t}-\pi_{t}^{*}$ represents the inflation gap, *y*_*t*_ represents the output gap, and β and γ are parameters. Considering the interest rate smoothing, we set the dynamic adjustment process of interest rate as follows:


(2) $$\begin{array}{l} i_{t}=\left(1-\rho \right)i_{t}^{*}+\rho i_{t-1}+e_{t} \end{array}$$

Where ρ ∈ [0, 1] represents the smoothing parameter of nominal interest rate, which means that the central bank makes partial adjustments based on the target interest rate and the previous interest rate level, which can eliminate the deviation between the current interest rate and the target interest rate. Thus, ρ represents that the central bank tries to make small adjustments to the interest rate, which will avoid changing the direction of interest rate frequently and causing large fluctuations in the economy. In addition, *e*_*t*_ is the random perturbation term. Therefore, by combining Equation (1) with Equation (2), it can be obtained:


(3) $$i_{t}=(1-\rho )[{\bar{i}}_{0}+\beta (\pi_{t}-\pi_{t}^{*})+\gamma y_{t}]+\rho i_{t-1}+e_{t}$$

Equation (3) is the specific form of the central bank's monetary policy rule with the characteristics of interest rate smoothing. It shows that the response of short-term nominal interest rate to the inflation gap and output gap depends on the magnitude and sign of β and, and then reflects the central bank's monetary policy adjustment mode.

The above Taylor rule indicates that in order to achieve the equilibrium state of the economic system, the central bank should adjust the nominal interest rate according to the output gap and the inflation gap. However, Taylor rule is an empirical formula based on the history of US monetary policy operation, which has obvious national attributes and stage characteristics, and may be distorted in matching with the reality of China. During the Southeast Asian financial crisis in 1997, China's monetary policy shifted in time, which effectively alleviated the problem of insufficient demand. After the outbreak of SARS in 2003, China implemented a prudent monetary policy and maintained its stability and continuity. From 2003 to 2007, during the period of rapid economic growth in China, the central bank continuously adjusted the deposit-reserve ratio, and the monetary policy changed from steady to tight. In response to the global financial crisis in 2008, China's monetary policy also responded quickly, changing from tightening to easing. At the same time, China launched supporting fiscal policies to stimulate the economy and release vitality, successfully realized the bottoming out of China's economic growth in 2009. In the post-crisis era, to cope with the substantial increase of money supply, the loose monetary policy gradually retreated. China's economic growth began to slow down in 2013 and the economy fully entered the new normal by 2015. The implementation of monetary policy has provided corresponding support for the economy. But since 2016, China's monetary policy has tightened again in line with the goal of “deleveraging”. The COVID-19 in 2020 brought a huge blow to the global economy. Macro-control is facing more complex and changeable circumstances, and the implementation of monetary policy is more difficult. However, the implementation of China's monetary policy played a role in suppressing economic fluctuations during this period. The COVID-19 pandemic in 2020 dealt a huge blow to the global economy. Macro-control is facing more complex and changeable circumstances, and the implementation of monetary policy is more difficult. In general, China's monetary policy is flexible and characterized by “leaning against the wind”. In order to better fit the actual operation of China's monetary policy, this paper further extends the traditional Taylor rule form:


(4) $$i_{t}=(1-\rho_{t})[{\bar{i}}_{0}+\beta_{t}(\pi_{t}-\pi_{t}^{*})+\gamma_{t}y_{t}]+\rho_{t}i_{t-1}+e_{t}$$

In Equation (4), β_*t*_, γ_*t*_, and ρ_*t*_, respectively represent the time-varying characteristics of the interest rate's response to inflation and output and the time-varying characteristics of the central bank's adjustment of the interest rate. The above is the construction of the time-varying Taylor rule model.

### SV-TVP-FAVAR Model

The paper uses the method of Korobilis (31) to construct a factor-augmented vector autoregressive model with time-varying coefficients and stochastic volatility. SV-TVP-FAVAR model is an extension based on VAR model. Compared with TVP-VAR model, SV-TVP-FAVAR model allows higher freedom. Therefore, macroeconomic data can be added into the model to make the estimation results more reliable. In addition, the variable coefficient and covariance in the FAVAR model are fixed in the sample period, while SV-TVP-FAVAR model can give them time-varying characteristics to better describe the changes in the impact process. Anyway, SV-TVP-FAVAR model loosens the constraint conditions and introduces settings for stochastic volatility, time-varying and factor augmented characteristics. It not only considers the characteristics of variables changing with time, but also includes many macroeconomic variables, so as to avoid the omission of important information in the system.

In order to study the preferences of monetary policy, a VAR model needs to be established:


(5) $$\begin{array}{l} Y_{t}=B_{1}Y_{t-1}+\cdots +B_{p}Y_{t-p}+v_{t} \end{array}$$

Where *Y*_*t*_ is a (*k* × 1) vector, *Y*_*t*−1_ ⋯ *Y*_*t*−*p*_ is the p-order lag term of *Y*_*t*_ and *v*^*t*^ ~ *N* (0,Ω) is the disturbance term. However, the number of parameters in a traditional VAR model is limited, generally not more than 20, which makes it difficult to conduct effective research on monetary policy. Therefore, it is necessary to a construct factor augmented vector autoregression (FAVAR) model on the basis of classical VAR model. Given this, a large number of macroeconomic variables can be incorporated into the model to solve the dimensionality problem in monetary policy research. On the other hand, in order to study the dynamic changes of variables, we can make the coefficient matrix of the factor-augmented vector autoregressive model change over time. Based on the above considerations, the TVP-FAVAR model construction is as follows:


(6) $$\begin{array}{l} Y_{t}=B_{1t}Y_{t-1}+\cdots +B_{pt}Y_{t-p}+v_{t} \end{array}$$

Where $Y_{t}^{{}^{\prime }}={\left[f_{t},g_{t}\right]}^{{}^{\prime }}$, and *f*_*t*_ is a (*k* × 1) vector of latent factors. *f*_*t*_ is obtained by effectively decomposing the n-dimensional observables *X*_*it*_. *X*_*it*_ reflects macroeconomic operation, and $k<<n.g_{t}=\left[i_{t},\pi_{t}-\pi_{t}^{*},y_{t}\right]$, including interest rate (*I*_*t*_), inflation gap ($\pi_{t}-\pi_{t}^{*}$) and output gap (*y*_*t*_). The coefficient of lag term *b*_*jt*_ is a (*k* + 3) coefficient matrix. The covariance matrix of the disturbance term also varies with time, which is *v*^*t*^ ~ *N* (0,Ω).

Each original observables *X*_*it*_, along with vector of latent factors *f*_*t*_ and observed variables*g*_*t*_, satisfies the following regression model with stochastic volatility:


(7) $$\left\{ \begin{array}{l} x_{it}={\tilde{\lambda }}_{i}^{f}f_{t}+{\tilde{\lambda }}_{i}^{g}g_{t}+u_{it}\\ u_{i}{}_{t}=\rho_{1t}u_{it-1}+...+\rho_{iq}u_{it-q}+\varepsilon_{it} \end{array}\right.$$

Where ${\tilde{\lambda }}_{i}^{f}$ is a (*n* × *k*) coefficient matrix and ${\tilde{\lambda }}_{i}^{g}$ is a (*n* × 3) coefficient matrix. Assuming that ε_*it*_ and *f*_*t*_ are not correlated and there is no autocorrelation, the above equation can be written in the following form:


(8) $$\begin{array}{l} x_{it}={\tilde{\lambda }}_{i}^{f}f_{t}+{\tilde{\lambda }}_{i}^{g}g_{t}+\Gamma \left(L\right)X_{t}+\varepsilon_{t} \end{array}$$

Where Γ(*L*) = *diag*(ρ^1^(*L*), …, ρ^*n*^(*L*)), $\rho^{i}\left(L\right)=\rho_{i1}L+\cdots +\rho_{i1}L_{q}$, *j* = *f, u*, and ε_*t*_ ~ *N*(0,*H*_*t*_) with *H*_*t*_ = *diag*(exp(*h*_1*t*_), …, exp(*h*_*nt*_)). *h*_*it*_ follows a driftless random walk of the form:$h_{it}=h_{it-1}+\eta_{t}^{h}$, $\eta_{t}^{h}\sim N\left(0,\sigma_{h}\right)$.

In the parameter estimation of the SV-TVP-FAVAR model, the method of two-steps estimation of Stock and Watson (32) is used for reference. First, the parameters of the unobservable part extracted from the background database are estimated, and then the (*K* × 1)-dimension vector obtained here together with other parameters in the model are performed to Bayesian estimation.

In order to avoid the problem of unknown sample distribution, the Gibbs sampling (33) is used to estimate the initial setting involved in the process, the prior probability density of the factor equation is $\left[\lambda_{i}^{f},\lambda_{i}^{g}\right]\tilde{\;}N\left(0_{1\times \left(k+i+1\right)},10I_{\left(k+i+1\right)}\right)$, $\Gamma_{i}\left(L\right)\tilde{\;}N\left(0_{1\times q},10I_{q}\right)$, $h_{i0}\tilde{\;}N\left(0,4\right)$, $\sigma_{h}^{-1}\tilde{\;}Gamma\left(0.01,0.01\right)$, where the prior probability density of the regression equation is $B_{0}\tilde{\;}N\left(\overset{⌢}{B},\overset{⌢}{V}\right)$, $C_{0}\tilde{\;}N\left(0,4I\right)$, $\log\sigma_{0}\tilde{\;}N\left(0,4I\right)$, $Q_{B}^{-1}\tilde{\;}W\left(0.005\times \left(\dim\left(B\right)+1\right)\times \overset{⌢}{V},\left(\dim\left(B\right)+1\right)\right)$, $Q_{C}^{-1}\tilde{\;}W\left(0.01\times \left(\dim\left(C\right)+1\right)\times I,\left(\dim\left(C\right)+1\right)\right)$, $Q_{\sigma }^{-1}\tilde{\;}W\left(0.0001\times \left(\dim\left(\sigma \right)+1\right)\times I,\left(\dim\left(\sigma \right)+1\right)\right)$, for B and C, dim(*B*) = *m* × *m* × *p*, dim(*C*) = *m*(*m* − 1)/2, respectively, forσ, dim(σ) = *m*, one-order lag coefficient $\overset{⌢}{B}=0.9$, other situations $\overset{⌢}{B}=0$.$\overset{⌢}{V}$ is a covariance diagonal matrix; the coefficients of lag term and variables are ${\overset{⌢}{V}}_{ij}=\frac{1}{c^{2}}$, respectively; lag order *c* = 1, …, *p*, stands for variance. $p\left(J_{t}^{\theta }=1\right)=\pi_{\theta }=1-p\left(J_{t}^{\theta }=0\right)$, $\pi_{\theta }\tilde{\;}Beta\left(1,1\right)$, *E*(π_θ_) = 0.5, *std*(π_θ_) ≅ 0.29, θ_*t*_ ∈ {*B*_*t*_, *C*_*t*_, logσ_*t*_}.

Finally, this paper mainly focuses on analyzing the three-dimensional dynamic response relationship between variables. The impulse response function can be set as follows:


(9) $$\begin{array}{l} \text{IR}{\text{F}}_{Y,K}\left(u_{it},\omega_{t-1}\right)=E\left[Y_{t+h}|u_{it},\omega_{t-1}\right]-E\left[Y_{t+h}|\omega_{t-1}\right] \end{array}$$

Where *E*[·] represents the expected operator, *h* represents the prediction length and *h* = 1, 2, 3⋯, *IRF*_*Y,K*_ represent the impact response function of variable *Y*, *u*_*i,t*_ is any information impact, and ω_*t*−1_ represents the historical information set of prediction *Y*.

## Variable Selection

### Monetary Policy Proxy Variables

The framework construction and estimation of time-varying parameter Taylor rule in this paper mainly involve variables including short-term nominal interest rate, inflation gap and output gap. We use data from the first quarter of 1996 to the third quarter of 2021 for modeling. The specific data are described in Table 1.

**Table 1:** Statistical description of the main variables in the article.

**Var**.	**Sam. Per**.	**Mean**	**Max**.	**Min**.	**Std. Dev**	***P*-Value**
GDP (%)	1995Q4–2021Q3	15.6034	28.9467	8.0333	4.8182	0.9343
Diff (GDP)	1996Q1–2021Q3	−0.0002	0.0547	−0.0257	0.0125	0.0000
I (%)	1995Q4–2021Q3	3.6410	12.5567	1.01	2.5405	0.0921
Diff (I)	1996Q1–2021Q3	−0.0013	0.4508	−0.8246	0.2669	0.0137
Inf (%)	1995Q4–2021Q3	2.7544	22.600	−2.1667	3.7678	0.6929
Diff (Inf)	1996Q1–2021Q3	−0.0828	2.500	−3.1333	0.0982	0.0000

*M2 represents broad measure of money supply; I represents the weighted average 7-day inter-bank offered rate; Inf represents inflation; Diff represents first order difference of original data; Var., variable; Sam. Per. represents sample period; Max., maximum; Min., minimum; Std. Dev., standard deviation*.

#### The Selection and Processing of the Short-Term Nominal Interest Rate

In countries with interest rate liberalization, the short-term nominal interest rate is the operational indicator of monetary policy. However, China's current interest rate system has not fully realized marketization, market interest rate and regulated interest rate coexist, so it is necessary to select a proxy variable that meets the requirements of market interest rate. Interbank lending and bond repurchase are both important components of the interbank market. The interbank lending market established in the first quarter of 1996 can most sensitively reflect short-term liquidity changes in the market and is one of the markets with the highest degree of marketization. Moreover, compared with the bond repurchase market rate, the interbank offered rate can better reflect the supply and demand situation in the money market. Therefore, we refer to some classic literature (34, 35) and take the weighted average 7-day inter-bank offered rate as the proxy variable of short-term nominal interest rate. The data source is the People's Bank of China Quarterly Statistical Bulletin.

#### Selection and Processing of the Inflation gap

Because the consumer price reflects the final price formed by the commodity through all links of circulation, it reflects the demand for money for the commodity circulation in the most comprehensive way. Therefore, CPI is the price index that can most fully and comprehensively reflect inflation. In this paper, CPI is selected as the proxy variable of inflation. The data can be obtained from the website of National Bureau of Statistics of China. In addition, since China does not implement the inflation targeting system, we draw on the practices of relevant classical literature (34) and set an inflation target of 4%. Therefore, this paper first converts monthly CPI data into quarterly data through arithmetic average, and the inflation gap π_*t*_ = (*CPI* − 100)/100 × 100% − 4%.

#### Selection and Processing of the Output gap

As there are differences in the measurement methods of potential output, the estimation of the output gap will also be different. In this case, the reliability of the research results would be compromised. Therefore, this paper uses wavelet filtering, singular spectrum analysis, production function method and Hodrick-Prescott filter method to measure the output gap in China. In the sampling interval, the data obtained by the four methods are consistent on the whole. However, in the special period corresponding to economic emergencies at home and abroad, the fluctuation characteristics are different. After analysis and comparison, the wavelet filtering method can capture the important economic nodes more accurately. Therefore, this paper chooses the wavelet filtering method to measure the output gap.

### Macroeconomic Variables Background Database

In order to describe the dynamic relationships among variables effectively, it is necessary to reduce the problem of information omission in the economic system. This paper sets the first quarter of 1996 to the third quarter of 2021 as the sampling interval. In this interval, we select a total of 78 original variables from the real economy, financial activities and prices to extract unobservable common factors, which can comprehensively reflect the operation of the macroeconomy. Specifically, the three levels are as follows: (1) The level of the real economy, including GDP, consumption, investment, and so on; (2) Financial activities, including various interest rates, stock indexes, and so on; (3) Price level, including CPI, PPI, and so on.

## Empirical Results

### Dynamic Relationships Among Variables

**Figure 1:**
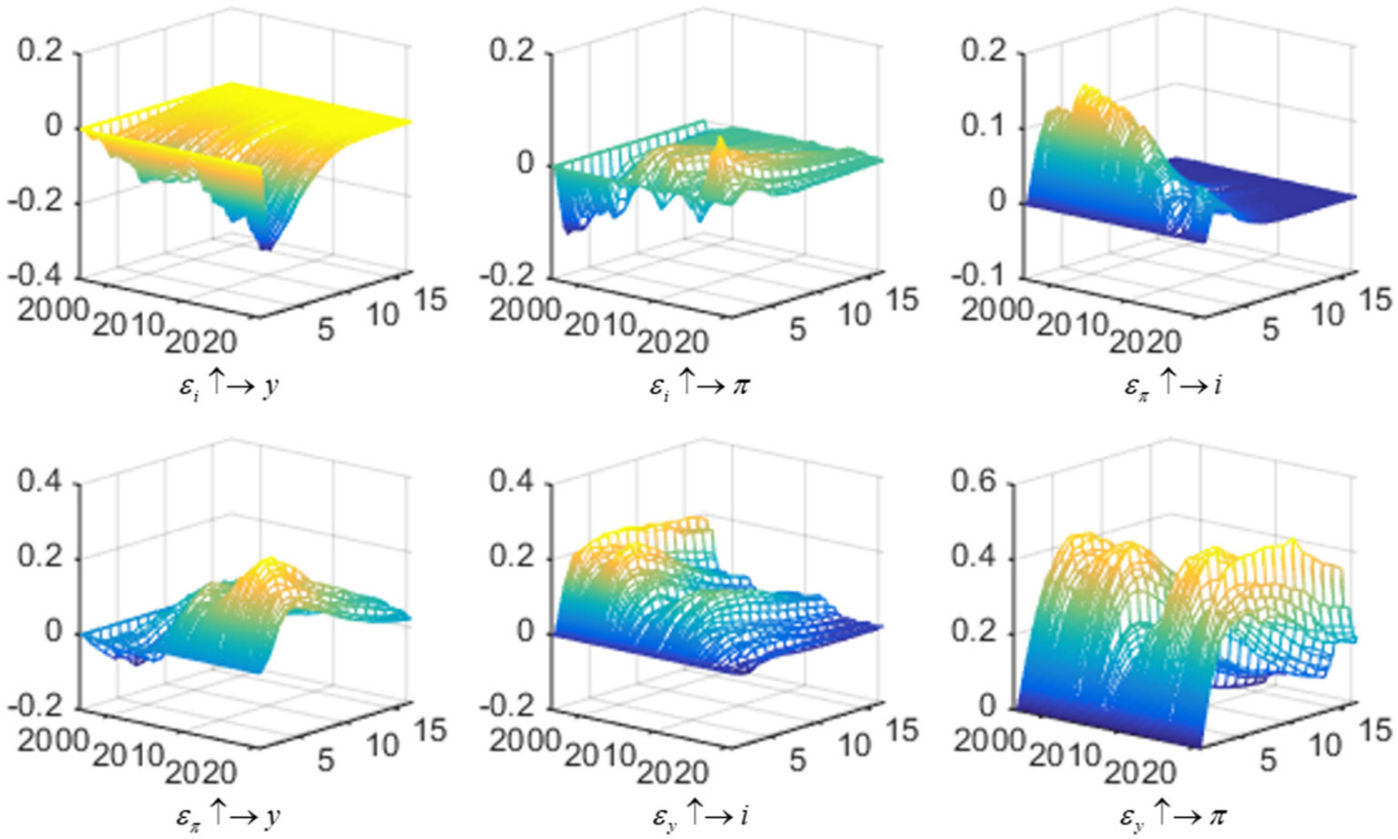
Three-dimensional impulse response of relationships among three variables of China's monetary policy. Each subfigure with the title of “*X ↑*→*Y*” demonstrates the response of variable Y to an orthogonalized positive shock to variable X. In other words, X is an impulse variable, and Y is a response variable. Three variables include interest rate (*i*), inflation gap (π), and output gap (*y*). One period in the figure denotes one season.

Based on the constructed SV-TVP-FAVAR model, this part deeply investigates the interaction among interest rate, output gap, and inflation gap within the sampling interval. Three-dimensional images of the impulse response functions above show the dynamic relationships among variables. The sampling interval is set from the first quarter of 1996 to the third quarter of 2021. Figure 1 shows three-dimensional impulse response images of relationships among three variables of China's monetary policy.

Firstly, the response of the output gap to interest rate shock (ε_*i*_ ↑→ *y*) and inflation gap (ε_π_ ↑→ *y*) shock. In terms of the time dimension from 1996 to 2021, the impact of interest rate shock on the output gap was negative for most of the time, and this negative response showed a gradually increasing trend. This result shows that the increase of interest rate will inhibit investment and consumption demand, thus leading to a negative deviation between actual output and potential output. Similarly, the decrease of interest rate will stimulate demand and lead to a positive change of output gap. Moreover, with the deepening of interest rate liberalization reform in recent years, the transmission mechanism becomes more smooth, so the fluctuation of the output gap caused by the change of interest rate is greater, and the correlation between interest rate and output is stronger. From the perspective of the time dimension, the response of the output gap to the inflation gap can be harmful in the early stage; the latter is positive. This trend of first falling and then rising is because the impact of the inflation gap on the output gap changes the effect of endogenous variables and exogenous reasons such as the expansion of fiscal policy and changes in complex economic situations at home abroad. Regarding the duration of shock-response, the impact of the interest rate on the output gap is mainly short-term. Generally, the extreme value of the shock response occurs after two quarters and gradually approaches zero after 2 years. At the same time, the impact of the inflation gap on the output gap approaches zero after 5 years. Regarding the level of shock—response, the output gap fluctuates more concerning the inflation gap than the interest rate.

Secondly, the response of the inflation gap to interest rate shock (ε_*i*_ ↑→ π) and output gap (ε_*y*_ ↑→ π) shock. Before 2006, interest rate shock had an adverse reaction to inflation gap. It shows that raising interest rates can reduce market liquidity and withdraw money, thus effectively curbing inflation. There was a gradual succession of positive and negative effects in the later period, influenced by fiscal policy, structural adjustment, and other measures. The adjustment of monetary policy is less effective in restraining the inflation gap. In addition, the impact of the output gap on inflation shows a positive response relationship. This main reason is that the positive change of output gap generally represents overcapacity, which leads to the increase of money supply in society, and thus the rise of price level leads to inflation. From the perspective of duration, the impact of interest rate on inflation peaks after two quarters and decreases to zero after 3 years. The effect of the output gap on inflation tends to zero in 5 years, and it takes longer to moderate from 2012 to 2020. Compared to the impact of the interest rate on inflation, the effect of the output gap on inflation is more significant from the level of response.

Finally, the response of the interest rate to inflation gap shock (ε_π_ ↑→ *i*) and output gap shock (ε_*y*_ ↑→ *i*). The interest rate positively to both inflation and the output gap. This means that when the inflation gap or the output gap increases, the interest rate will be improved accordingly. The root cause is that the inflation gap is generally manifested as excess liquid. The reacts the positive impact of the output gap also suggests that spare capacity at this time, the central bank can adjust the interest rate to tame the inflation and output gaps. The above analysis is consistent with the theory of the Taylor rule. From the response dimension, this positive response shows a decreasing trend. Precisely, the interest rate can well capture the running track of the Chinese economy in response to changes in the inflation gap and output gap. The interest rate for adjusting the output gap and inflation gap presented a downtrend before the Asian financial crisis in 1997. In 2008, the global financial crisis triggered by the subprime mortgage crisis in the United States had an impact on economy. So the output gap decreased, accompanied by a certain degree of deflation. China mainly adopted a loose monetary policy to stimulate economic recovery by raising the interest rate during this period. To get rid of the impact of the financial crisis, China adopted the “four trillion” stimulus plan in 2009, which effectively increased the demand and caused overcapacity, economic instability, and other problems. This series of problems may increase financial risks, so the central bank chose to raise the interest rate to control. Since 2012, China has been in a period of economic new normal. The impact response of interest rate to the output gap has been gradually reduced, the fluctuation is relatively gentle, and the adjustment range is also tiny in COVID-19. The interest rate in response to inflation shocks is broadly similar to the analysis above. However, since the bottom of 2017, there has been another recovery trend, indicating that the current economy is stabilizing, and the main goal of monetary policy is to regulate and control inflation. Regarding the duration of the response, the response of interest rate to the output gap and inflation gap peaks in the sixth and second quarters, respectively, and then approaches zero five and 2 years later. From the overall level of response, the volatility of interest rates caused by output gap shock is more significant than that caused by the inflation gap.

Through the above analysis, we can conclude that the correlation among interest rate, output gap, and inflation gap exhibits significant time-varying and non-linear characteristics, which indicates that the time-varying Taylor rule is more consistent with the actual trend of China's monetary policy. In addition, the impulse effect between variables is mainly reflected in the short and medium-term, which also confirms that the Taylor rule mentioned above is a short-term interest rate adjustment policy based on the inflation gap and the output gap. Furthermore, inflation and output gap impact the interest rate and have a positive effect, indicating that China's monetary policy prefers targeting inflation and output gaps. But over time, this preference has gradually weakened. It shows that the regulatory effect of China's monetary policy on interest rate and output gap is gradually weakening. In a specific period, the preference of monetary policy will show asymmetric characteristics, which will be explained in the next section.

### Dynamic Relationships Among Variables in Special Periods

**Figure 2:**
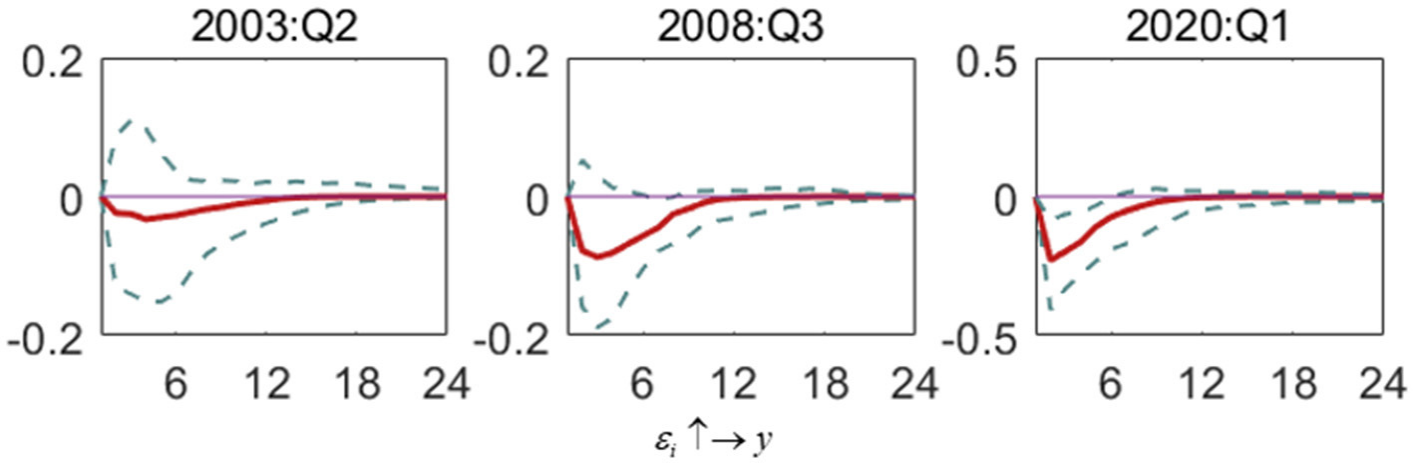
The impulse response of the output gap to interest rate shock in special periods. Each subfigure demonstrates three special periods, including the SARS outbreak period (2003:Q2), the global financial crisis period (2008:Q3) and the COVID-19 outbreak period (2020:Q1). One period in the figure denotes one season.

**Figure 3:**
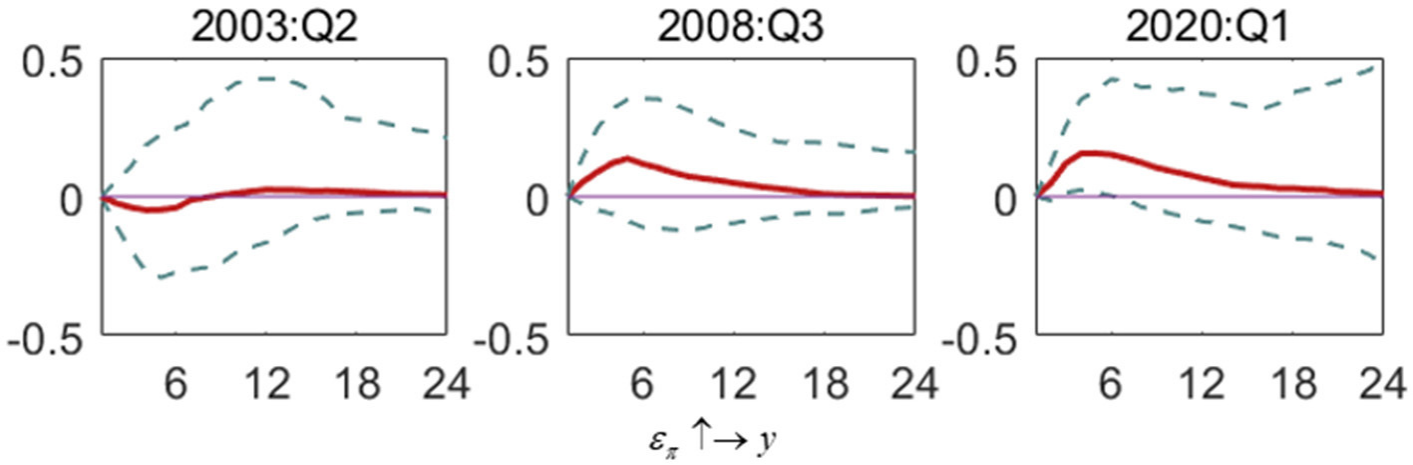
The impulse response of the output gap to inflation gap shock in special periods. Each subfigure demonstrates three special periods, including the SARS outbreak period (2003:Q2), the global financial crisis period (2008:Q3) and the COVID-19 outbreak period (2020:Q1). One period in the figure denotes one season.

**Figure 4:**
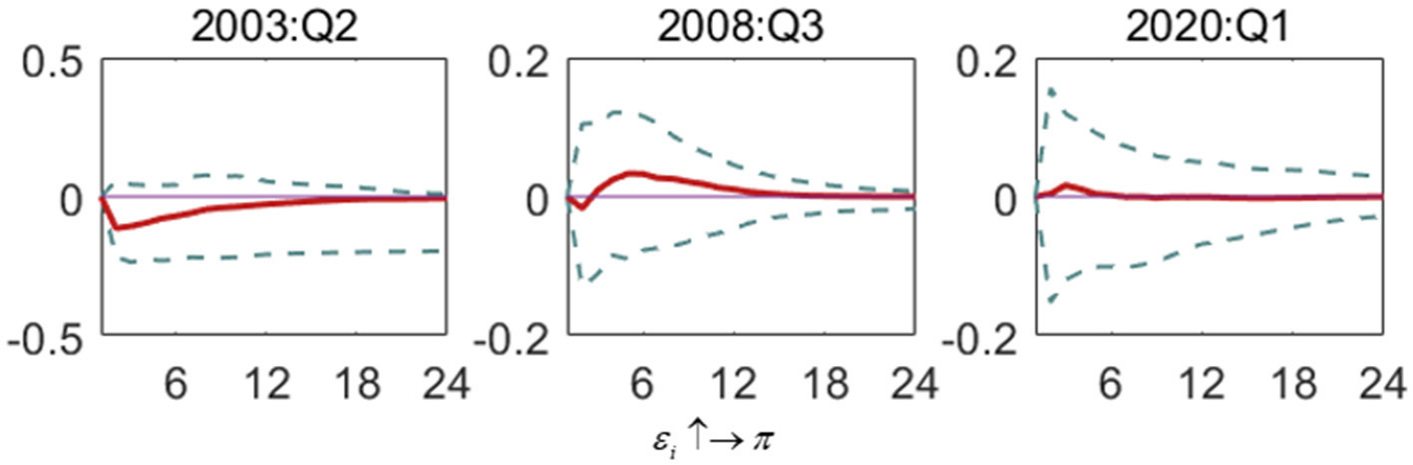
The impulse response of the inflation gap to interest rate shock in special periods. Each subfigure demonstrates three special periods, including the SARS outbreak period (2003:Q2), the global financial crisis period (2008:Q3) and the COVID-19 outbreak period (2020:Q1). One period in the figure denotes one season.

**Figure 5:**
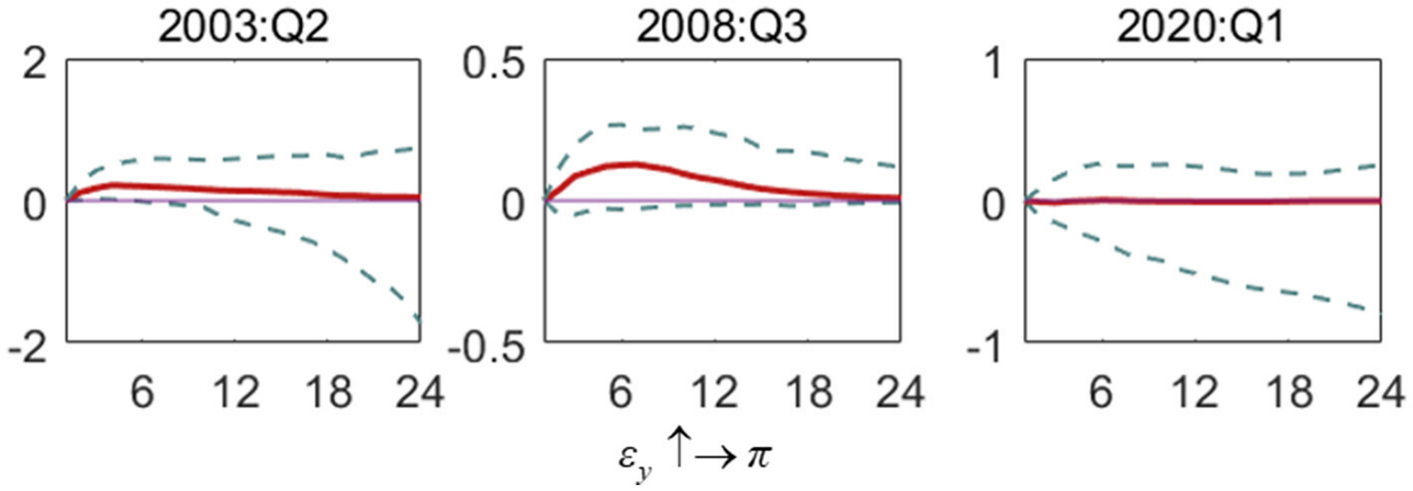
The impulse response of the inflation gap to output gap shock in special periods. Each subfigure demonstrates three special periods, including the SARS outbreak period (2003:Q2), the global financial crisis period (2008:Q3) and the COVID-19 outbreak period (2020:Q1). One period in the figure denotes one season.

**Figure 6:**
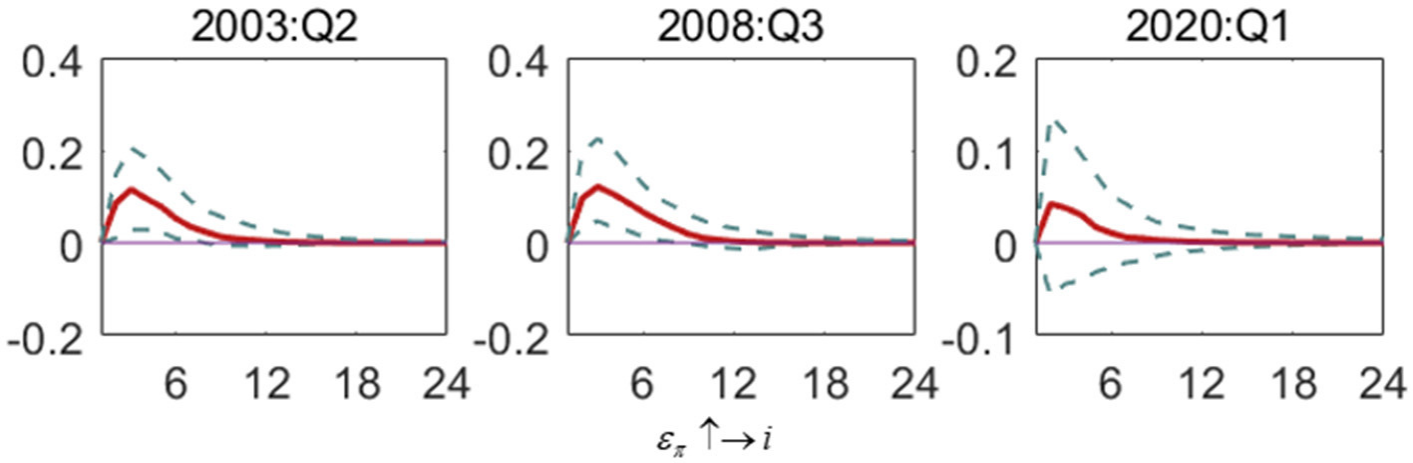
The impulse response of the interest rate to inflation gap shock in special periods. Each subfigure demonstrates three special periods, including the SARS outbreak period (2003:Q2), the global financial crisis period (2008:Q3) and the COVID-19 outbreak period (2020:Q1). One period in the figure denotes one season.

**Figure 7:**
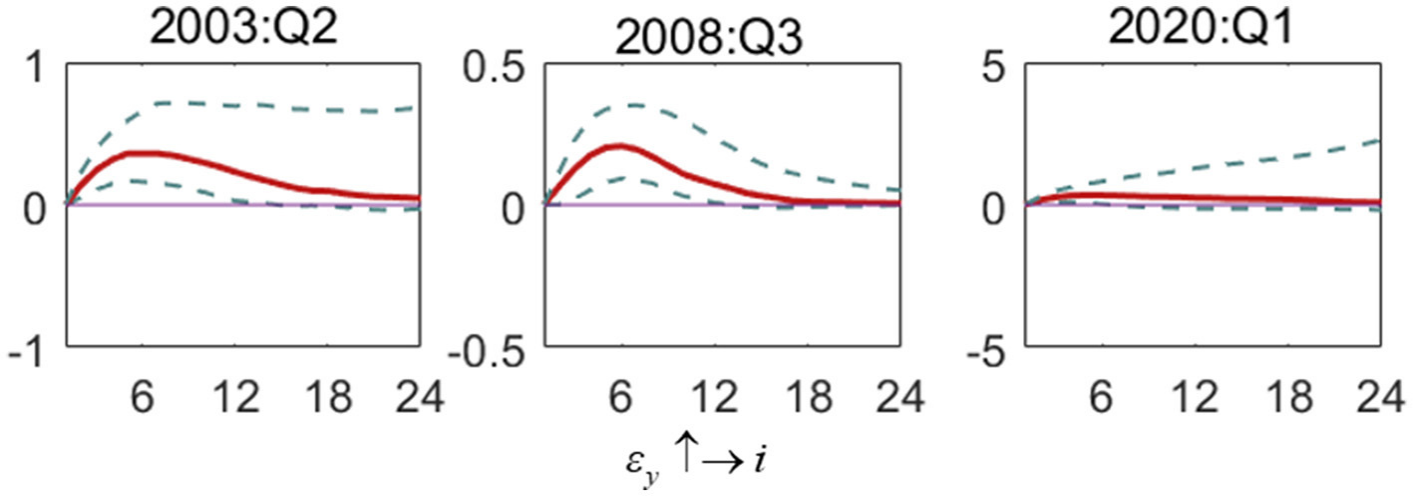
The impulse response of the interest rate to output gap shock in special periods. Each subfigure demonstrates three special periods, including the SARS outbreak period (2003:Q2), the global financial crisis period (2008:Q3) and the COVID-19 outbreak period (2020:Q1). One period in the figure denotes one season.

This paper selects the first quarter of 1996 to the third quarter of 2021 as the sample period to examine the mutual feedback effect among interest rate, output gap, and inflation gap before and after the epidemic. By comparing the impulse response functions before and after the epidemic, we comprehensively analyze the orientation of monetary policy, that is, the adjustment path and time-varying characteristics of monetary policy to the output and inflation gaps. After having an overall understanding of relationships among interest rate, inflation gap, and the output gap, the part focuses on the new characteristics of the role of monetary policy in the context of the COVID-19 pandemic. At the same time, the SARS outbreak period (the second quarter of 2003) and the global financial crisis period (the third quarter of 2008) within the sampling range are selected as the comparison to further elaborate the changes in China's monetary policy preferences during the crisis periods, especially after the outbreak of COVID-19. Figures 2–**7** demonstrate three-dimensional impulse response of relationships among three variables of China's monetary policy in special periods.

Figure 2 shows the impact of interest rate shock on the output gap (ε_*i*_ ↑→ *y*). In general, the output gap negatively responds to interest rate shock during a crisis, indicating that interest rate adjustment will lead to economic instability during an emergency. Among them, during the outbreak of the COVID-19, the interest rate shock leads to the most significant fluctuation in the output gap, but it also converges at a faster rate. In contrast, the degree of volatility is more minor during the financial crisis, and convergence speed is also slower. During the SARS outbreak, the fluctuation of the output gap caused by the interest rate shock is the smallest, and the response and recovery time is the longest. This shows that the outbreak of COVID-19 has a vast and unexpected impact on the economy. Hence, the output gap responds to the adjustment of interest rate to a large extent in the first quarter of 2020. However, the fluctuation quickly recovers due to the state's effective prevention and control measures and a series of policies to stabilize the economy.

Figure 3 shows the impact of inflation gap shock on the output gap (ε_π_ ↑→ *y*). In the SARS period, inflation gap shock causes the negative influence of the output gap. But with time, the impact turns to be negative. During the financial crisis and the COVID-19 outbreak, the influence of the inflation gap on the output gap is positive. At the same time, the volatility of the two is relatively sim; that is, they are both more significant than the volatility of the output gap caused by the inflation gap during the SARS period. It shows that the financial crisis and the outbreak of the COVID-19 have a wider scope, leading to significant changes in inflation, which will have a greater impact on the social economy. The peak of the output gap's response to inflation gap changes during the COVID-19 and financial crisis appears in the third and fifth periods, respectively, and the recovery of the response during the COVID-19 outbreak takes longer than that during the financial crisis.

Figure 4 shows the impact of interest rate shock on the inflation gap (ε_*i*_ ↑→ π). Interest rate fluctuations affect the inflation gap in different special periods. During the SARS period, changes in interest rates lead to a negative response to the inflation gap, indicating that the increase in interest rates can effectively suppress the inflation gap. However, during the financial crisis, the effect turns from negative to positive, producing a positive impact during COVID-19. The SARS period has the highest volatility in response amplitude and slows down relatively slowly, followed by the financial crisis period. The COVID-19 period has the lowest volatility, with the response degree approaching zero after the fifth period. This shows that in recent years in the economic downturn, the interest rate as a tool for monetary policy control has weakened its impact on inflation, which requires other such as a fiscal policy to assist or solve the problem from the source of the imbalance between supply and demand.

Figure 5 shows the impact of the output gap shock on the inflation gap (ε_*y*_ ↑→ π). The fluctuation of the output gap has an apparent positive effect on the inflation gap in different periods. The output gap has the most significant impact on the inflation gap in the financial crisis period, and the response range gradually approaches zero after 16 periods. In the SARS period, the output gap responds less to the inflation gap than in the financial crisis period. In the COVID-19 era, the output gap is the least volatile in terms of shocks caused by the inflation gap. It can be seen that the increase of output gap fluctuation will increase the inflation gap. Still, in the post-epidemic era, the impact of the output gap on inflation is lower than in the previous period.

Figure 6 shows the impact of inflation gap shock on the interest rate (ε_π_ ↑→ *i*). In the above three special periods, inflation changes will cause a positive reaction of interest rate, which indicates that interest rate will increase with the increase of inflation gap. China's monetary policy has the preference feature of controlling the inflation gap. Compared with SARS and the financial crisis, the interest rate response to the inflation shock during COVID-19 is weaker. The maximum response of the former two exceeds 0.1, but the full responsibility of the COVID-19 period is only around 0.04, and the recovery time of the shock is also shorter. This suggests that in COVID-19, the moderating effect of the interest rate on the inflation gap has weakened. This may be because the central bank has been implementing loose monetary policy for a long time, which has reduced the space for monetary policy regulation. On the other hand, it also shows that China is better able to deal with crisis events. In response to the impact of COVID-19, the reverse regulation mode of monetary policy is less aggressive and more prudent.

Figure 7 shows the impact of the output gap shock on the interest rate (ε_*y*_ ↑→ *i*). In the SARS period and the financial crisis period, the fluctuation impact of the output gap will lead to a positive response of interest rate, showing the preference of monetary policy to target the output gap. However, in the COVID-19 period, the impact of interest rate on output gap impact shows an alternating positive and negative trend, and the effect is not significant. In addition, compared with the financial crisis and SARS, the shock response during the COVID-19 outbreak has been very small, fluctuating near zero. In short, compared with previous special periods, during the COVID-19 period, the interest rate is no longer subject to drastic adjustment with changes in the output gap, and the functional space of monetary policy to stimulate the economy in response to the crisis is constantly reduced. In addition, compared with the impact of interest rate on inflation in the same period, China's monetary policy still prefers to control the inflation gap, and the scope of interest rate adjustment focusing on the output gap is relatively small. Thus, compared with economic instability, the central bank pays more attention to the excessive fluctuation of inflation.

## Conclusion

This paper sets the first quarter of 1996 to the third quarter of 2021 as the sampling interval. By constructing the SV-TVP-FAVAR model, we estimate relationships among the variables contained in the Taylor rule. In view of this, we choose three special crisis periods to compare, and further study the preference characteristics and implicit monetary policy information of the central bank in the COVID-19 era. According to a series of three-dimensional impulse response function images, there are dynamic correlations among the interest rate, the output gap, and the inflation gap. The specific conclusions are as follows. First, the output gap has an obvious characteristic of adjusting to the impact of the interest rate and the inflation gap. During the period of COVID-19, the impact of interest rate shock on the output gap is significantly negative, indicating that the adjustment of China's interest rate policy in response to COVID-19 can stimulate investment and consumer demand, thereby contributing to the effective allocation of social resources, so as to achieve the goal of stabilizing the economy. Meanwhile, the impact of inflation gap shock on output gap is positive, which is not only caused by the change of endogenous variables, but also caused by exogenous reasons such as the expansion of fiscal policy and the change of complex economic situation at home and abroad. Second, the impact of inflation gap on interest rate shows alternating positive and negative effects in stages, but this impact has gradually diminished in recent years. It indicates that the adjustment of monetary policy has weakened the inhibitory effect on inflation, and fiscal policy needs to be assisted or solve the problem from the root of supply-demand imbalance. In addition, the inflation gap shows a significant positive response to the impact of the output gap. Compared with other crisis periods, this effect also shows a trend of narrowing in the COVID-19. Third, generally speaking, the interest rate has the characteristics of targeting the output gap and the inflation gap in the short term. When the inflation or output gap increases, the interest rate will rise correspondingly, which indicates that China's monetary policy follows the non-linear time-varying Taylor rule. In addition, in terms of time trend, in recent years, especially after the outbreak of COVID-19, the impact of inflation gap and output gap on interest rate has shown a declining trend, indicating that the regulation of China's monetary policy on inflation and output gap has weakened. It also shows that the monetary policy has learned from the experience of coping with crises in the past, the system is more mature, and its regulation mode is more moderate and accurate. At the same time, the response coefficients of interest rate to the impact of inflation and output gap are different. Compared with targeting the output gap, monetary policy has more obvious preference to control inflation. It can be seen that there is a trade-off between economic growth and price stability. When the crisis occurs, relevant departments in China pay more attention to the stability of the output gap. However, after the crisis, due to the excessive issuance of money, there will usually be large-scale inflation, and the focus of the monetary authority will shift to stabilize prices. Since the economic new normal, the intensity of regulation between monetary policy and inflation gap has decreased, which suggests that the government should timely consider promoting economic growth while paying attention to price stability. In addition, the inflation gap shows a significant positive response to the impact of the output gap. Compared with other crisis periods, the effect also shows a narrowing trend in the COVID-19. It indicates that there is still much room for improvement in the implementation of monetary policy. The government should pay attention to promoting the rational combination of monetary policies using different tools according to the economic objectives in different periods, which can improve the regulation efficiency of policies and avoid the conflict of policy objectives. Finally, the results of the time-varying parameter model show that it is very important to explore the non-linear dynamic effects in the implementation of China's monetary policy, which can provide corresponding decision-making basis for the monetary authorities on the implementation of monetary policy and the adjustment of time-varying objectives.

The above research results show that the interest rate, inflation gap, and output gap are significantly correlated, so regulators should pay attention to the interactive characteristics of the three. It contributes to understand the internal mechanism of monetary policy implementation. Secondly, monetary policy orientation is real-time and asymmetric. In the post-epidemic era, the economic situation is complex and changeable. Hence, paying attention to macroeconomic changes and the timeliness of monetary policy can provide an important basis for the central bank to formulate monetary policy and adjust the time-varying target. At present, with the effective control of the global epidemic, inflation has become a new challenge for economic recovery. Therefore, the implementation of monetary policy needs to further enhance its flexibility and moderation rather than radical operation. The government needs to grasp the rhythm of economic operation, pay attention to the inflation target, give full play to the regulatory role of monetary policy and maintain the overall stability of price level. Finally, according to the results of the analysis, China's monetary policy regulation space has the trend of relative reduction. Therefore, it is more need to improve the robustness, flexibility and accuracy of monetary policy. At the same time, the government can also use fiscal policies, such as taxes, subsidies and other financial means, to more accurately solve the structural imbalance caused by the COVID-19 to the economy. The central bank of China can also use innovative monetary policy tools such as MLF to dredge the transmission channels of the interest rate. It can realize the effective coordination between conventional and unconventional monetary policies, and then deal with the impact of the COVID-19.

## Data Availability Statement

The original contributions presented in the study are included in the article/supplementary material, further inquiries can be directed to the corresponding author/s.

## Author Contributions

ZH: conceptualization, methodology, software, formal analysis, data curation, writing—original draft preparation, writing—review, and editing. BZ, SW, and ZH: validation. BZ: investigation, resources, supervision, project administration, and funding acquisition. SW: visualization. All authors contributed to the article and approved the submitted version.

## Funding

This research was funded by the Key Project of National Social Science Foundation (Grant No. 20AZD043), the Key Project of Chinese Ministry of Education (Grant No. 17JZD016), and the National Natural Science Foundation of China (Grant No. 11901233).

## Conflict of Interest

The authors declare that the research was conducted in the absence of any commercial or financial relationships that could be construed as a potential conflict of interest.

## Publisher's Note

All claims expressed in this article are solely those of the authors and do not necessarily represent those of their affiliated organizations, or those of the publisher, the editors and the reviewers. Any product that may be evaluated in this article, or claim that may be made by its manufacturer, is not guaranteed or endorsed by the publisher.

## Abbreviations

WHO: World Health Organization
IMF: International monetary fund
$i_{t}^{*}$: short term nominal interest rate included in the basic Taylor rule
ī_0_: long-term and time-varying equilibrium interest rate
$\pi_{t}-\pi_{t}^{*}$: inflation gap
*y*_*t*_: output gap
*i*_*t*_: short term nominal interest rate considering the interest rate smoothing
CPI: consumer price index
GDP: gross domestic product
PPI: producer price index
MLF: medium-term lending facility.
